# Using alt text to make science Twitter more accessible for people with visual impairments

**DOI:** 10.1038/s41467-020-19640-w

**Published:** 2020-11-16

**Authors:** Domenico Chiarella, Justin Yarbrough, Christopher A.-L. Jackson

**Affiliations:** 1 Clastic Sedimentology Investigation (CSI), Department of Earth Sciences, Royal Holloway, University of London, London, UK; 2 Digital Accessibility Specialist, Instructional Media and Design, Rio Salado College, Tempe, AZ USA; 3 Basin Research Group (BRG), Department of Earth Science & Engineering, Imperial College, London, UK

**Keywords:** Geology, Ethics

## Abstract

Scientists increasingly post images and photos on social media to share their research activities. However, posting images and photos could potentially exclude people with visual impairments. Here, we outline actions that should be taken to foster accessibility and inclusion in posting scientific images on social media.

Social media is all around us. It is a truly global method of communication, with an estimated 3.5 billion users worldwide. Twitter, Instagram, and Facebook allow us to update friends and followers on the challenges and successes of our research activities, as well as our daily lives. Twitter has become the social media platform most commonly used by academics to share research and rapidly discuss scientific analysis between themselves, as well as with the general public around the globe^1–3^. These tools allow academics to escape the time-constraints imposed by the journal-based publishing method of communicating results, allowing faster dissemination to a far broader audience, potentially increasing the impact and reach of our research^4^. But with this comes a responsibility to ensure that the content we publish is accessible to all.

Social media is particularly important for the natural sciences community. Natural sciences data are very visual and benefit from the use of videos, images, photos, and animated GIFs, functionality readily available on most social media platforms. Image use strongly benefits field-based areas of natural sciences, be that at the top of a mountain or in the depths of the oceans. To maximise and broaden education and research experiences, it is important that any shared material is accessible to all social media users, many of whom are typically overloaded with information from both academic and non-academic sources. Image-rich social media is thus a potentially key, yet currently underutilised tool for education and research in most field-based disciplines.

## Barriers

At the same time, the very action of posting images and photos could exclude those with visual impairments who use screen reading software to access social media. Gleason et al.^5^ analysed over nine million tweets (including both original tweets and retweets) collected across five days from all publicly-available Twitter profiles (not specifically identified as scientist or non-scientists), finding that about 1.09 million (11.84%) contained an image, and that only 1144 (0.1%) of those included an image description. Scientists have the responsibility to foster accessibility on social media, and a good practice would be to include a description to posted images related to research outcomes.

Field-based disciplines are often perceived as being ‘closed’ to people with disabilities^6–8^. This is because field sites may be inaccessible, or because it is considered important to have visual contact with the actual rock or landscape in order to describe and understand it. Recent studies^7,9–11^ propose actions like the use of audio field guides and tactile maps to foster inclusion and accessibility and overcame some of these barriers so that natural sciences programmes are accessible for everyone. Image-rich social media thus represents an additional potential way to bring the field to those interested in the natural sciences.

Social media tools have introduced accessibility features such as the ability to modify colour contrast, adjust the text size, and add descriptions to graphics. However, a recent study shows that people with vision impairment face profound accessibility challenges when dealing with graphics without sufficient text description^12^. Nevertheless, people with visual impairments interact with photos posted on social media as often as people without these impairments, although they notably share fewer photos than the average social media user^12,13^.

## Actions to take

Alternative text (also known as alt text), a feature now offered on many social media platforms, can be used to provide a more inclusive experience for people with visual impairments when sharing image-rich content. It allows users to add a text-based alternative (usually a description) to non-textual content such as photos, GIFs and graphical data, although the nature of graphical data provides some limitation on the use of the alt text. And Alt text is not currently available for video content. A user’s screen-reading software, through speech output, will have the alt text presented to them in place of the graphic. Users with combined hearing and vision loss may instead use a screen reader in conjunction with a refreshable braille display, which mechanically raises and lowers “dots” mimicking printed braille; this allows them to read screen reader output.

To achieve our collective mission of making the world more open and connected, we have to reach out to people of all backgrounds and abilities. As scientists, it is our duty to make every effort to foster accessibility and inclusion in our disciplines, which are often perceived as containing numerous barriers to engagement due to their strong link with field-based activities. The simple action of adding a clear and concise description of a graphic posted on social media makes a significant positive impact on users with visual impairments. Although exhaustive descriptions may be problematic due to the limited number of characters available (e.g., 280 characters in the body of a single Twitter post), Twitter has developed a feature allowing users to add descriptions to static images as alt text—as of May 2020, alt text is enabled by default on the web as well as iOS and Android apps meaning that no longer a setting is needed to add image descriptions. A complete list of best practices on how to increase social media accessibility is available at https://accessibility.umn.edu/tutorials/accessible-social-media.

**Fig. 1:**
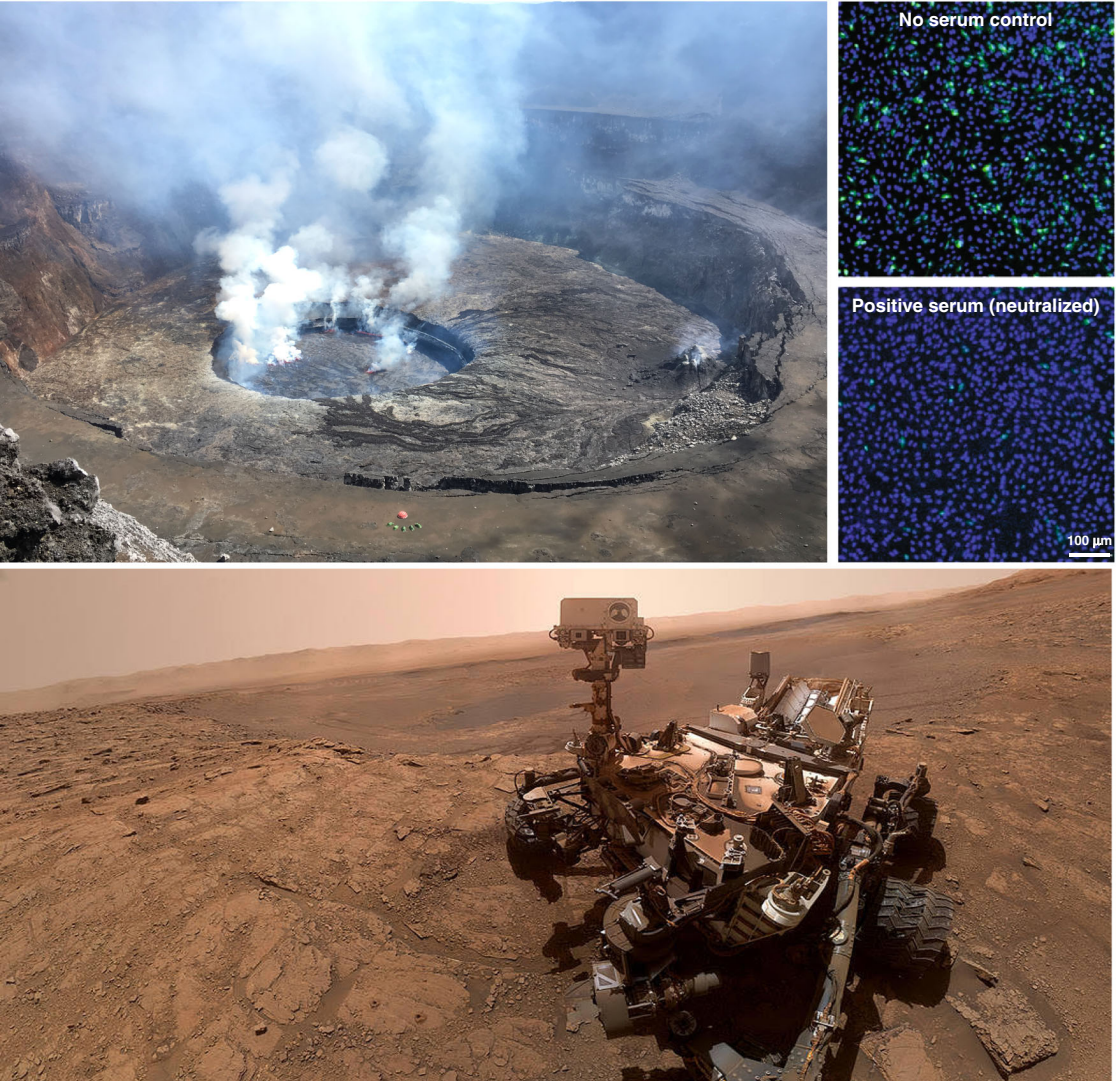
Image description (alt text) example for different scientific images. On the left, a giant hole in the ground at the top of the Nyiragongo volcano (Democratic Republic of Congo) containing a black lava lake that is approximately 200 metres in diameter. There are whitish gas plumes rising from holes in the semi-solid surface of the lake. The holes are glowing orange. There are six tents only a few hundreds of metres away from the lava lake. On the right, Vero CCL-81 cells used in a fluorescence-based assay designed to measure neutralisation of a reporter SARS-CoV-2 by antibodies from patient specimens. After reporter viral infection, the cells turned green in the absence of serum (top panel). In contrast, incubation of the reporter virus with COVID-19 patient serum decreased the number of fluorescent cells (bottom panel)^14^. Scale bar, 100 μm. On the bottom, selfie taken by the NASA’s Curiosity rover on the 11^th^ October 2019 in the Glen Etive location (Gale Crater, Mars) showing the rover and two holes “Glen Etive 1” and “Glen Etive 2” drilled alongside it (Credits: NASA/JPL-Caltech).

While the content of image descriptions will vary from person to person, a few key points should be considered when composing alt text. Keep in mind the purpose of the image and focus on describing the elements that further that purpose. When possible, avoid redundancies between image descriptions and the text of the tweet; e.g. avoid the use of phrases like “graphic shows” and “photo of,” since assistive technologies will identify the content as a graphic on their own. Do not include content such as links in alt text, since users of assistive technology cannot easily access the links. Instead, place links in the text of the tweet. Lastly, be as concise as possible with your descriptions (e.g., Fig. 1). Twitter limits alt text to 1000 characters per image (https://help.twitter.com/en/using-twitter/picture-descriptions) and some older versions of assistive technology struggle with lengthy alt text.

Fostering inclusion and accessibility is key in many areas of our society, and every action should be promoted to reduce or preferably remove barriers. Social media represents a tool to reduce the experience gap for people with visual impairments. Recent innovations in artificial intelligence have resulted in the ability to generate image descriptions automatically (e.g., Twitter A11y^13^). That being said, it should be noted that “automatic” alt text should not be relied upon as an alternative, as it will likely either misidentify objects or not provide an appropriate level of detail in descriptions, especially for scientific purposes. The best is to sensitise the scientific community to the needs of those with visual impairments, and to foster accessibility and inclusion.
